# Macrophage: A Potential Target on Cartilage Regeneration

**DOI:** 10.3389/fimmu.2020.00111

**Published:** 2020-02-11

**Authors:** Tiago Lazzaretti Fernandes, Andreas H. Gomoll, Christian Lattermann, Arnaldo Jose Hernandez, Daniela Franco Bueno, Mariane Tami Amano

**Affiliations:** ^1^Sports Medicine Division, Institute of Orthopedics and Traumatology, Hospital das Clínicas HCFMUSP, Faculdade de Medicina, Universidade de São Paulo, São Paulo, Brazil; ^2^Hospital Sírio-Libanês, São Paulo, Brazil; ^3^Department of Orthopedic Surgery, Center for Cartilage Repair and Sports Medicine, Brigham and Women's Hospital, Harvard Medical School, Boston, MA, United States; ^4^Hospital for Special Surgery, New York, NY, United States

**Keywords:** M1/M2 macrophages, cartilage regeneration, synovial inflammation, mesenchymal stem cells, osteoarthritis, articular cartilage, cell therapy

## Abstract

Cartilage lesions and osteoarthritis (OA) presents an ever-increasing clinical and socioeconomic burden. Synovial inflammation and articular inflammatory environment are the key factor for chondrocytes apoptosis and hypertrophy, ectopic bone formation and OA progression. To effectively treat OA, it is critical to develop a drug that skews inflammation toward a pro-chondrogenic microenvironment. In this narrative and critical review, we aim to see the potential use of immune cells modulation or cell therapy as therapeutic alternatives to OA patients. Macrophages are immune cells that are present in synovial lining, with different roles depending on their subtypes. These cells can polarize to pro-inflammatory (M1) and anti-inflammatory (M2) phenotypes, being the latter associated with wound-healing by the production of ARG-1 and pro-chondrogenic cytokines, such as IL-10, IL-1RA, and TGF-b. Emerging evidence reveals that macrophage shift can be determined by several stimuli, apart from the conventional *in vitro* IL-4, IL-13, and IL-10. Evidences show the potential of physical exercise to induce type 2 response, favoring M2 polarization. Moreover, macrophages in contact with oxLDL have effect on the production of anabolic mediators as TGF-b. In the same direction, type II collagen, that plays a critical role in development and maturation process of chondrocytes, can also induce M2 macrophages, increasing TGF-b. The mTOR pathway activation in macrophages was shown to be able to polarize macrophages *in vitro*, though further studies are required. The possibility to use mesenchymal stem cells (MSCs) in cartilage restoration have a more concrete literature, besides, MSCs also have the capability to induce M2 macrophages. In the other direction, M1 polarized macrophages inhibit the proliferation and viability of MSCs and impair their ability to immunosuppress the environment, preventing cartilage repair. Therefore, even though MSCs therapeutic researches advances, other sources of M2 polarization are attractive issues, and further studies will contribute to the possibility to manipulate this polarization and to use it as a therapeutic approach in OA patients.

## Introduction

Cartilage repair is the critical issue that patients with symptomatic cartilage lesions seek (1). Chondral lesion is a pathology with high prevalence, reaching as much as 63% of general population and 36% among athletes (2, 3). It has an impact in socioeconomic health system and the attempted treatment of these lesions is associated with a considerable economic burden (4). For instance, cartilage and osteoarthritis treatment can delay joint replacement and improve symptoms (5).

Articular cartilage tissue presents limited cellularity and lacks a vascular system, leading to restrained healing capability (6, 7). Actually, there is no available treatment to regenerate hyaline cartilage or modify disease progression (8). Consequently, cartilage injuries are often related to pain and joint instability that may diminish or even cease the tissue's functionality (6, 7). Thus, articular cartilage is at high risk of damage during initial trauma and, if left untreated, may results in lesions in the underlying subchondral bone, leading to biomechanics and homeostasis disturbances in the knee as a whole. This process may result in loss of mobility, wear and arthritis (9, 10).

Despite the numerous techniques available today, complete healing of damaged or defective cartilage and the consistent reproduction of normal hyaline cartilage is an elusive goal (5). For these reasons, continuous drug therapies and secondary surgeries are common, and new therapeutics for articular cartilage lesions is of elevated clinical relevance (11, 12).

Among cell therapeutics solutions, it is observed two main examples: Autologous Chondrocyte Implantation (ACI) and Mesenchymal Stem Cells (MSCs).

ACI is a two-step procedure that consists of healthy cartilage harvesting through arthroscopy followed by the expanded cell culture and, in a second step, cartilage defect filling (13–15). In spite of second and third ACI generations' versatility, those techniques use healthy cartilage tissue, “*in vitro*” related chondrocytes dedifferentiation and still fails to fully reproduce the hyaline characteristics of the original articular cartilage (6, 13, 15–17).

More recently, mesenchymal stem cell-based therapy has received considerable attention, because of the feasibility of handling the tissue harvest and *ex vivo* cell expansion and differentiation (12, 13). Moreover, these cells present minor immunological rejection due to the low surface expression of major histocompatibility complex (MHC) antigens, efficient engraftment and long-term coexistence in the host, which turns them an attractive therapeutic option (18, 19). According to the International Society of Cell Therapy (ISCT), MSCs are defined as plastic-adherent when maintained in standard culture conditions, specific surface antigen expression, and the cells must be able to differentiate to osteoblasts, adipocytes and chondroblasts under standard “*in vitro*” differentiating conditions (20).

The capacity of allogeneic MSCs to repair cartilage lesions has been reported in clinical trials (21) and in translational large animal models (12, 22). These cells migrate to damaged tissues and contribute to their repair by secretion of cytokines, chemokines, and extracellular matrix proteins (23). The chondrogenic potential and the known immunosuppressive characteristics of MSCs, point out these cells as a powerful tool in the therapy of osteoarthritis (OA) (18).

## Articular Cartilage And Inflammation

The estimated prevalence OA in the population is 22.7% and it is believed that by 2020, more than 50 million people will suffer from OA in the United States. This will be the major cause of morbidity and physical limitation among individuals aged over 40 years (24). Current therapy for OA is directed toward non-pharmacological treatments as physical activity through mechanical stimulation (25) and symptomatic treatment, focusing in pain management, and is not able to promote regeneration of degenerated cartilage or to attenuate joint inflammation (18).

A cartilage breakdown results in a release of molecular fragments into synovial fluid that starts macrophages fragment removal in the synovium and further signaling in a positive feedback loop triggering apoptosis in chondrocytes (26).

OA is a disease which affects all joint tissues, characterized by progressive degeneration of the articular cartilage, neovascular invasion of articular surface, subchondral bone remodeling, osteophyte formation, bone marrow lesions, meniscal damage and synovial inflammation (synovitis) (18, 27). Joint effusion is detected in half of patients with OA symptoms and no radiographic findings, indicating that synovitis is not restricted to severe OA only, but is associated with increased pain and dysfunction (28).

Accumulating evidence suggests that synovial inflammation is correlative with the pathogenesis and progression of OA (27). And articular inflammatory environment is the key factor for initiation and aggregation of cartilage lesion (29). Clinical symptoms of OA are attributed to synovial inflammation (26).

Synovial inflammation is characterized by synovial thickening with hypertrophy and hyperplasia (30). Increased vascular density and inflammatory cell infiltration (lymphocytes and macrophages) are common features of OA (31). Macrophage accumulation in the intimal lining, reflecting mostly proliferative synovial tissue, is the principal morphological characteristic of synovitis (27).

A substantial part of OA patients develops synovial activation (32). Synovial lining macrophages play a crucial role in driving joint pathology, such as cartilage damage and ectopic bone formation (32). Macrophage-derived inflammatory cytokines [tumor necrosis factor alpha (TNF-a) and interleukin (IL)-1b] shift synovial tissue homeostasis toward catabolism by promoting production of matrix degrading enzymes that results with an increased bone and cartilage resorption (18). In a rat model of OA induced by anterior cruciate ligament transection, it was also observed increased of inflammatory OA-related cytokines, such as TNF-a, IL-1b, and matrix metallopeptidase 13 (MMP13). While moderate physical activity decreased the expression of these cytokines and increased the level of anti-inflammatory and chondroprotective proteins, such as IL-4 and IL-10 (25) corroborating the idea of immune response contributing to the synovial dynamic.

The synovial membrane is an area of high functional importance within the joint, responsible for the production of synovial fluid, which lubricates and nourishes chondrocytes (31). The membrane is composed of two cell types, synovial macrophages and fibroblasts (31). Normal synovium consists of two distinct tissue layers. One is the intimal lining layer with two to three layers of macrophages (type A cells) with phagocytic function and fibroblast-like synoviocytes (type B cells) with secretory function that produces hyaluronan and lubricin (25, 26); the other is the synovial sublining layer, composed of fibrous connective tissue and blood vessels, with few lymphocytes or macrophages (27).

OA development involves multiple pathological changes, including synoviocytes dysfunction, chondrocytes apoptosis and hypertrophy and immune cells activation (25, 33). These changes have an impact in the articular microenvironment, being crucial for the cartilage repair (33). Labinsky et al. (34) stated that OA inflammation is not homogeneous, different inflammatory phenotypes exists and it may influence each patient selection criteria and treatment (28). Thus, the OA treatment requires a multifactorial approach that restrain inflammatory response and provide a pro-chondrogenic microenvironment. Macrophages are protagonists in the balance of inflammation and regeneration, which catches the attention for the possibility of their use as therapeutic tools.

## Macrophages

Macrophages, together with fibroblasts, are present in the synovial lining of joints. They are involved in synovial inflammation, and have been shown to play a prominent role in the progression of OA (27). Macrophages are innate immune cells that express MHC class II, which gives them the ability to initiate adaptive immune response through T cell activation. Macrophage from synovial tissue expresses CD14, CD68, CSF1R, HLA-DRA, and MARCO (35).

After initial stimulation, macrophages acquire a phenotype, ranging from pro-inflammatory (M1) to anti-inflammatory (M2) (36). In the joint, CD14 and CD163 are associated to pro-inflammatory and anti-inflammatory phenotypes, respectively (28). Synovial macrophages can have different origins, being embryonic- or bone marrow-derived, and depending on the source they might present different roles in arthritis (37). Culemann et al. demonstrated a dynamic membrane-like structure formed by resident synovial CX3CR1^+^ macrophages that physically isolate the joint and restrict inflammation. These CX3CR1^+^ macrophages are derived from mononuclear cells embedded in the synovial tissue, and present opposite function from infiltrating monocyte-derived macrophages, which are responsible to inflammatory response in the joint (38).

These data suggest a therapeutic potential of the modulation of macrophages in OA.

### Macrophage M1

Macrophages M1 were seen to be associated to Th1 response. Mice strains that were known to favor Th1 (C57Bl6) or Th2 (BALB/c) responses, presented preferentially induction to M1 or M2 differentiation, respectively (39). M1 macrophages are the “classically activated” and are associated with high production of pro-inflammatory cytokines and chemokines as shown in Table 1 (27, 40). M1 macrophages can be induced by interferon-g (IFN-g) and lipopolysaccharide (LPS) increasing CD80 expression (41). Lepage et al. also stated that monocytes subjected to TNF-a can polarize to M1 phenotype (36). Once in contact with these stimuli, transcription factors, such as IRF5 activates transcription of genes encoding IL-12, IL-23 and represses IL-10 (42). M1 stimulation also leads to nitric oxide (NO) production and cell surface expression of the co-stimulatory molecules CD86, CD80, and MHC, which are required for T cell activation (43). In general, M1 macrophages have high microbicidal activity and secrete large amounts of pro-inflammatory cytokines (Table 1) (36, 44).

**Table 1:** Macrophage polarization in OA and cartilage regeneration.

**Macrophage polarization**	**Stimuli**	**Transcription factors**	**Phenotype**	**Released products**	**Functional roles**	**Potential tools for OA treatment**
M1	IFN-g, LPS, TNF-a	IRF1, IRF5, IRF8, Pu.1, STAT1, STAT2, NFkB	CD80, CD86, CD40, MHC-II	TNF-a, IL-1b, IL-6, IL-12, IL-23, OSM, NO, CXCL10, IL-8, CCL5, CXCL9, CXCL11, MMP1, MMP3, MMP9, MMP13, ADAMTS	Inflammation, type 1 response and tissue injury microbicidal. **→** **OA induction**.	M1 blocker^**^ Depletion of CD14^+^ Macrophages^**^
M2	IL-4, IL-13, IL-10 and TLRs agonists, MSCs (?)	IRF4, Pu.1, SOCS1, STAT6, JMJD3, PPARg, PPARd, GATA3, C/EBPb	ARG-1, CD163, CD206	IL-10, IL-1RA, TGF-b, IGF, CCL18, CCL4, CCL13, CCL17, MMP1, MMP12	Anti-inflammatory response, type 2 response, tissue repair, chondrogeneic and turnover of extracellular matrix. **→** **Cartilage regeneration**.	M2 inducers: oxLDL, collagen type II, MSCs^*^. Blocking mTOR pathway?^**^

* *The M2 polarization can be halt by the microenvironment*.

** *Further studies are required*.

*OA, osteoarthritis*.

*Bold is used to highlight information*.

### Macrophage M2

The “alternatively activated” macrophages were denominated M2, and they are known as wound-healing macrophages. The M2 macrophages have been further divided into specific subtypes: M2a (induced by IL-4 and IL-13), M2b (induced by TLRs agonists), and M2c (induced by IL-10). All subtypes promote anti-inflammatory responses (44). After M2 induction, the transcription factor IRF4 among others are activated favoring the polarization (45) (Table 1). M2 macrophages are characterized by the expression of CD163 and CD206 markers and production of arginase (ARG)-1 (Table 1). These cells present an anti-inflammatory function producing, IL-10, IL-1RA, chemokine (CeC motif) ligand 18 (CCL18), and TGF-b (31, 46) as well as pro-chondrogenic factors: TGF-b1, TGF-b2, TGF-b3, insulin-like growth factor 1 (IGF1), and 2 (IGF2) (33) (Table 1). The IL-10-induced subtype M2c plays a role in tissue remodeling (36). Some of the known M2-related genes are *ARG-1*, resistin like alpha (*FIZZ1*), mannose receptor, C type 1 (*MRC*), human macrophage galactose-type C-type lectin (*CLEC10A*) (47). Arginine metabolism into nitric oxide (NO) and citrulline (M1 macrophages) or ornithine and urea (M2 macrophages) may be used to distinguish phenotypes. Their relative proportion of NO/urea is useful for functional readout since it reflects the ratio of M1/M2 polarization (8).

## Macrophages In OA

Macrophages play pivotal roles in innate immunity and exhibit a high degree of plasticity. Synovial macrophages have similar phenotype than others resident macrophages, including CD11b, CD14, CD16, and CD68 (30). O'Brien et al. (26) found that there were more macrophages in the early stages of synovial OA than compared to the late stages. They also demonstrated that synovial macrophages are decreased in pre-OA joints in comparison to normal knees, and that MSCs and macrophages are spatially closer to each other in normal and pre-OA than in OA cases (26). M1 affects OA cartilage by inhibiting genes associated with matrix production, upregulation of matrix degenerating genes and induction of inflammation. Fahy et al. (31) stated that the M1-associated cytokines IL-6, IL1b, TNF-a, and Oncostatin M (OSM) induce destructive processes in chondrocytes including down regulation of collagen type II and aggrecan synthesis. Synovial M1 macrophages were also shown to up regulate the production of proteolytic enzymes, such as matrix metalloproteinase (MMP)-1, MMP3, MMP13, MMP9 aggrecanases (ADAMTS), and cyclooxygenase-2, which contribute to articular degeneration (8, 30, 31). It was demonstrated that synovial macrophages and monocyte-derived pro-inflammatory macrophages negatively affected chondrogenesis of MSCs (36).

**Figure 1:**
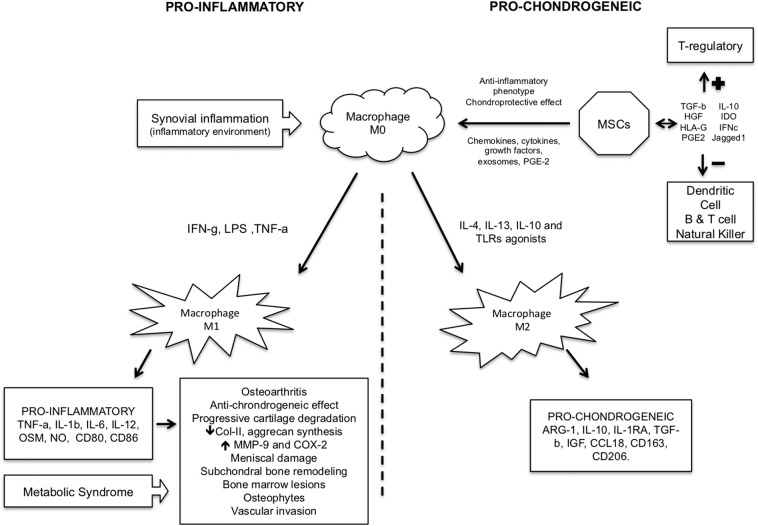
Pro-inflammatory and pro-chondrogeneic macrophage pathways in cartilage injury/repair.

M2 macrophages have a major role in tissue repair (44), and the shift from M0 toward M2 macrophages in the lesion might contribute to repair the damaged articular cartilage. Dai et al. showed that under the stimulation of certain biomaterials M2 macrophages could be induced, releasing certain regulatory cytokines and exerting an immunomodulatory effect on tissue healing (33) (Figure 1).

It seems clear that pro-inflammatory macrophage M1 contributes to OA, while M2 could reverse it or favor the chondrogenesis process. There are evidences showing that moderate physical activity could change the synovial microenvironment, shifting it from a type 1 to type 2 immune response, which is associated to M2 macrophages with the presence of IL-4 and IL-10 cytokines. These changes could provide a protective environment in the joints of OA patients (25). The use of gold nanoparticles were shown to present anti-inflammatory macrophage response (48), and a pilot study was opened as clinical trial (NCT03389906) in order to see impact of these particles in macrophages from OA knees, though no further analysis concerning M1 and M2 was mentioned.

Lepage et al. showed that M1 and M2a macrophages did not affect OA cartilage, while M2c seemed to induce inflammation to some extent, although less intense than M1. Both M2a and M2c were unable to counteract the pro-inflammatory effects of M1 (36). This was somewhat unexpected since it was previously demonstrated that M2 macrophages induced by macrophage colony-stimulating factor (M-CSF) enhanced chondrogenesis *in vitro* (36). The analogous M1 polarization to M2 M-CSF-induced is the stimulation with granulocyte macrophage colony-stimulating fator (GM-CSF) (49). The use of anti-GM-CSF was applied in a clinical trial with patients with OA. Although some positive effect was seen in diminishing the pain, MRI images showed no change in synovitis after anti-GM-CSF treatment (50). Wood et al. (35) declared that sub-groups of synovial macrophages functions as a spectrum with different remodeling signatures and Tardito et al. (51) emphasized the need of a not classical sub-division into M1 and M2 macrophages, but a M1 and M2 coexistence and equilibrium.

To date, there are few clinical studies in humans considering macrophage polarization and osteoarthritis (27, 34, 50, 52). Most of them do not access macrophage polarization and when they access, they are *in vitro* studies with human cells. It points out the novelty and clinical relevance of this topic, and the possibility for new clinical perspectives and treatments for OA.

Therefore, the M2 induction is crucial for chondrogenesis development, and inhibiting M1 macrophage appears to be a good target for treatments. However, further investigations are required before translating these approaches to the patients.

## Macrophage Modulation As Potential Tools For OA Treatment

### Macrophages and Oxidized LDL

Metabolic syndrome. is a combination of pathological processes that increases cardiovascular risks associating hypertension, diabetes, obesity and high levels of low-density lipoproteins (LDL). And it was reported to be associated to OA, though the mechanisms involved remain unclear (32, 53). Oxidized LDL (oxLDL) is a modified LDL and it interacts with macrophages that accumulate in the subendothelial space and transform into foam cells (54). This accumulation of cells leads to chronic inflammation in the arterial wall culminating in atherosclerosis initiation (54, 55). Despite the inflammation observed by foam cells, macrophages treated with oxLDL polarize to M2 phenotype, presenting increased production of IL-10 and TGFb (56).

de Munter et al. and Griffin et al. had postulated that low grade inflammation during OA could induce local oxidation of LDL aggravating OA pathology (57). Synovial fluid (SF) contains LDL (28, 58). Either serum- or serum free (SF)-derived LDL could be oxidized under inflammatory conditions and taken up by synovial cells (59). However, the same group demonstrated later that injections of oxLDL in knee joints in mouse model significantly increased TGF-b without inducing catabolic response and inflammatory response (32). Curiously, injection of oxLDL in macrophage-depleted animals increased inflammation, with higher expression of CCL2 and CCL3, attracting more monocytes and neutrophils and increasing synovial thickening (32). These data suggest that oxLDL injections could contribute to OA protection by inducing M2 macrophages.

### Macrophages and Collagen Type II

Type II collagen has been classically recognized as the indispensable collagenous component in articular cartilage, and plays a critical role in the development and maturation process of chondrocytes (33). *In vitro* studies reported that type II collagen increases secretion of cartilage matrix by chondrocytes (60).

M2 macrophages express mannose receptors MRC1 (CD206) and together with MRC2 can recognize several types of collagen, promoting internalization and lysosomal degradation (61, 62). M2 macrophages were shown to contribute to collagen turnover to keep the extracellular matrix homeostasis (63). On the other hand, type II collagen was shown to induced M2 polarization, by increasing the expression of M2-related genes (MR, Arg1, Fizz1, and Ym1) as well as the pro-chondrogenic cytokines (TGF-b and IGF). In an OA rat model, one of the groups was treated with collagen type II injections and it was observed increased M2 macrophages, higher production of TGF-b in synovial fluid, diminished chondrocyte apoptosis and decreased MMP13 production (33). MMP13 degrades cartilage matrix (type II collagen and proteoglycan) (64).

Since M2-associated cytokines participate in tissue repair (65), we could infer that collagen type II could favor M2 polarization, turning the microenvironment prone to chondrogenesis, contributing to cartilage repair and keeping the extracellular matrix stability.

### Macrophages and mTOR Pathway

Emerging evidence reveals that the mammalian target of rapamycin (mTOR) pathway plays key roles in macrophage polarization (27). The canonical activation of this pathway is by the phosphorylation of PI3K, followed by AKT that phosphorylate the tuberous sclerosis complex (TSC1-TSC2-TBC1D7) (66). The TSC2 phosphorylated inhibits the Ras homolog enriched in brain (Rheb), which in turn activates mTORC1. The downstream activation of this pathway leads to changes in metabolism that favor cellular growth signals, modulating innate and adaptive immune response (67). Genetically modified animals with TSC specifically depleted from myeloid cells (TSC^−/−^), which therefore leads to constitutive mTOR complex 1 (mTORC1) activation, were shown to favor M1 and fail to polarize to M2 phenotype (68, 69). In a low synovial activation OA model, the TSC^−/−^ mice presented increased M1 accumulation in synovial and exacerbated experimental OA (27). Conversely, deletion of Rheb1 in the myeloid lineage (Rheb1^−/−^) enhanced synovial macrophage M2 polarization and attenuated OA (27).

The production of IL-12, iNOS, and TNF-a (M1-like macrophage markers) are upregulated in TSC1^−/−^ macrophages in comparison to control macrophages, indicating the M1 inflammatory phenotype with mTORC1 activation (68). Taken together, these findings demonstrate that M1-polarized macrophages induced by mTORC1 promote hypertrophic chondrocyte differentiation and maturation, suggesting that it plays a critical role in cartilage degeneration during OA. The inhibition of mTOR pathway has long been used in transplanted patients (with drugs, such as rapamycin, sirolimus, and everolimus), because of its property of blocking T cell proliferation (70), one could consider the use of these inhibitors to treat OA. However, Zhu et al. demonstrated that inhibition of mTOR did not reverse the M1 response in TSC^−/−^ cells (69). Moreover, PI3K knockout (71) or mTORC2 deleted macrophages (72), both presenting impaired mTOR pathway activation, also favored M1 polarization, showing a complex scenario before considering a therapeutic approach by manipulating this pathway.

### Macrophages and Mesenchymal Stem Cells

It is well-known that MSCs exhibit immune-tolerance capacity by downregulating effector immune cells response and favoring an immunosuppressed environment. MSCs can influence innate immune cells, such as macrophages, dendritic cells, and natural killer cells, as well as adaptive immune cells, such as T and B lymphocytes (23, 73, 74). One of the mechanisms that MSCs can influence immune cells is through secretion of immune regulating molecules, such as TGFb, hepatocyte growth factor (HGF), HLA-G, prostaglandin (PGE2), IL-10, and indoleamine 2,3-dioxygenase (IDO) (23, 73). Besides the paracrine secretion of cytokines, MSC can also modulate inflammatory response by cell to cell (46).

MSCs inhibit activation of inflammatory M1 macrophages and promote anti-inflammatory M2 polarization *in vitro* (18). MSCs is associated to the conversion of TNF-a and IL-1 inflammatory cytokines into immunosuppressive IL-10 production by macrophages, which attenuate joint inflammation and promote cartilage regeneration (18).

CD14 is a membrane antigen (glycoprotein) expressed on the surface on macrophages and monocytes (52). In the other sense of immune-modulation, Han et al. stated that depletion of human CD14^+^ synovial macrophages allows osteoarthritic synovial MSCs for chondrogenic potential (52). NF-kB represents a family of inducible dimeric transcription factors that stimulates osteoarthritis and it is a pivotal factor that induces suppression of the chondrogenic potential of human osteoarthritic synovial MSCs (52). Chahal et al. (75) demonstrated a reduced level of monocytes/macrophages pro-inflammatory IL-12 cytokine in synovial fluid levels and likely improved clinical efficacy in patient-reported outcomes after 3 months of high doses of MSCs injection in the knee (75).

It was demonstrated that intra-articular injection of adipose derived stromal cells (ASCs) in OA animal models exert anti-inflammatory and chondroprotective effects (76, 77). In cell culture, MSCs secrete a large number of chemokines, cytokines, and growth factors that pushes macrophages to polarize toward an anti-inflammatory phenotype (46).

Hamilton et al. demonstrated a decrease in proportion of iNOS and reduction of pro-inflammatory macrophages after MSCs injection in a murine OA model (78). It was reported that MSCs decreased synovial inflammation and fibrosis (78).

PGE2 is a lipid mediator derived from the conversion of arachidonic acid to the prostaglandin through COX1 and COX2 enzymes that has an important role in MSC immunosuppression. PGE2 production by MSCs promote conversion of M1 to M2 phenotype (79). Manferdini et al. demonstrated that PGE2, mainly produced by ASC, was directly responsible for inhibition of the inflammatory cytokines TNF-a and IL6 (46) and blocking PGE2 by EP4 receptor antagonist showed the opposite effect corroborating these data (80).

Nevertheless, osteoarthritic-conditioned medium and synovial fluid were shown to inhibit the chondrogenic differentiation of MSCs (81, 82), which indicates that the presence of a destructive inflammatory environment, as found in OA, may halt the MSCs properties to cartilage repair. Fahy et al. showed that M1 macrophages inhibit chondrogenic differentiation of MSCs. These findings suggests that synovial macrophages are key regulators of the chondrogenesis of OA synovium (31).

Even though MSCs are clearly a potent M2 inducer, if the environment is exacerbated inflamed, MSCs by themselves may not be able to conduct the cells to type 2 response. Other stimuli, such as oxLDL, collagen type II, and signaling pathways manipulation could help to control the OA damage or cartilage repair.

## Final Considerations

Osteoarthritis is a disabling and very incapacitant disease with no definitive treatment that concerns patients and physicians. International societies are working hard on osteoarthritis treatment. Currently, no final or effective modifying-disease treatment is available and clinicians can only prescribe physical activity, alleviate symptoms or post-pone surgeries.

The clinical relevance of this study was to perform a critical review of literature and analysis of the current knowledge to open new roads for innovative and translational clinical trials of macrophage polarization in humans to treat osteoarthritis.

Macrophages plasticity can provide interesting therapeutic approaches in OA. There are several evidences that macrophage polarization could contribute to cartilage repair, either by inducing M2 or blocking M1 macrophages. M2 macrophages increase TGF-b, a well-known anti-inflammatory and pro-chondrogenic cytokine that contributes to MSCs differentiation and cartilage formation.

Since MSCs in an already inflamed tissue have their properties impaired, in addition to the attempts of the use of MSCs and their products (vesicles, micro-vesicles, and exosomes) in cartilage restoration, M2 macrophages induction could improve even more the MSCs therapeutic effects. Thus, further studies on M2 induction in combination with MSCs in cartilage repair could enormously contribute to OA treatments perspectives.

## Author Contributions

TF and MA took responsibility for the integrity of the study from conception and design to completion. Drafting of the manuscript and critical revision was performed by TF, MA, CL, AG, AH, and DB.

### Conflict of Interest

The authors declare that the research was conducted in the absence of any commercial or financial relationships that could be construed as a potential conflict of interest.
